# Safety and efficacy of laparoscopic sleeve gastrectomy in adolescents with Grade V obesity (BMI ≥ 50 kg/m^2^)

**DOI:** 10.1016/j.clinsp.2026.101017

**Published:** 2026-06-02

**Authors:** Haobing Guo, Guiqi Wang, Jian Zhang, Liyun Pang, Jingfeng Gu

**Affiliations:** Department of Gastroenterology Diagnosis and Treatment (Weight Loss and Metabolic Disease), The First Hospital of Hebei Medical University, Shijiazhuang, Hebei, China

**Keywords:** Laparoscopic sleeve gastrectomy, Adolescent, Grade IV obesity, Efficacy, Safety

## Abstract

**Objective:**

To investigate the effects of Laparoscopic Sleeve Gastrectomy (LSG) on the safety profile and metabolic parameter improvement in adolescents (10‒17 years-old) with grade IV obesity (BMI ≥ 50 kg/m^2^).

**Methods:**

From May 2023 to September 2024, a total of 60 adolescent patients with grade IV obesity who underwent LSG at the Department of Bariatric and Metabolic Diseases, The First Hospital of Hebei Medical University, were enrolled as the initial study cohort. Observations and statistics were collected on surgical-related complications, body weight, (Body Mass Index) BMI, glucose metabolism indicators, lipid metabolism indicators, liver function, and nutritional status to analyze the safety and efficacy of the procedure.

**Results:**

A total of 60 adolescent patients successfully underwent LSG without conversion to open surgery or perioperative mortality. During postoperative follow-up, only 31 patients completed regular assessments with complete data available for analysis. There were no perioperative period complications such as postoperative hemorrhage or gastric leak. Follow-up assessments at 3-months, 6-months, and 1-year postoperatively revealed significant improvements (*p* < 0.05) in the following parameters: body weight .[preoperative (154.1 ± 19.3) kg, 1-year postoperative (97.8 ± 13.6) kg], BMI .[preoperative (51.9 ± 3.8) kg/m^2^, 1-year postoperative (30.2 ± 1.4) kg/m^2^], uric acid .[preoperative (491.5 ± 92.4) μmoL/L, 1-year postoperative (376.8 ± 23.6) μmoL/L], liver function, blood lipids, glycated hemoglobin, and fasting insulin .[preoperative (57.9 ± 17.6) μIU/L, 1-year postoperative (15.5 ± 6.2) μIU/L] Preoperatively, 12 patients had hypertension, 8 had type 2 diabetes mellitus, 4 had Polycystic Ovary Syndrome (PCOS), and 10 had Non-Alcoholic Fatty Liver Disease (NAFLD). At 1-year after surgery, all patients with hypertension and type 2 diabetes mellitus achieved remission criteria. In addition, imaging findings and liver function improved significantly in 7 of the 10 patients with fatty liver disease, and clinical manifestations improved in 3 of the 4 patients with polycystic ovary syndrome. Concurrently, a declining trend (*p* < 0.05) was observed postoperatively in hemoglobin, trace elements (calcium, iron), and vitamins (Vitamin D, Vitamin B12), although all values remained within the normal range.

**Conclusion:**

LSG is a safe and effective procedure for improving metabolic disorders in adolescent patients with grade IV obesity, with confirmed short-term efficacy. However, the widespread declining trend in postoperative nutritional indicators underscores that systematic nutritional monitoring and intervention must be a core component of postoperative management in clinical practice to achieve long-term health benefits.

## Introduction

With the rising incidence of childhood and adolescent obesity and the increasing number of associated comorbidities, obesity can no longer be overlooked..^1^ It is estimated that people with obesity and its related complications impose a substantial economic burden: in the United States, the direct medical costs amount to approximately 170 billion annually; when indirect costs such as productivity losses are included, the total economic burden rises to 1.72 trillion.^2^ The pathogenesis of adolescent obesity is complex and multifactorial, leading to visceral fat accumulation and metabolic abnormalities. It may result in conditions such as obesity-related hypertension, type 2 diabetes, skeletal development issues, and even increased mortality.^3^^,^^4^ Obesity during childhood and adolescence is associated with a higher prevalence of cardiometabolic risk factors. Obesity in adolescents not only significantly elevates the risk of obesity in adulthood but is also closely linked to increased long-term all-cause mortality, as well as various psychological issues.^5^

Lifestyle intervention serves as the first step in the management of adolescents with grade Ⅳ obesity. For children over 12 years of age, pharmacotherapy constitutes the second step, and in specific circumstances, bariatric surgery is the third step.^6^ Lifestyle intervention does not yield long-term beneficial outcomes in adolescents with obesity. A randomized double-blind trial demonstrated that after 56 weeks of treatment, the proportion of patients achieving > 5% reduction in BMI was significantly higher in the liraglutide group compared with the placebo group (43.3%vs 18.7%); similarly, the proportion achieving exceeded 10% BMI reduction was also greater (26.1%vs 8.1%). However, the incidence of gastrointestinal adverse events was higher in the liraglutide group. Bariatric surgery is the most effective approach for treating people with grade Ⅳ obesity, with laparoscopic sleeve gastrectomy (LSG) being the most widely adopted and well-established surgical procedure.^7^ The American Academy of Pediatrics recognized LSG as an effective treatment for adolescents with grade II or III obesity accompanied by severe complications.^8^^,^^9^ The proportion of adolescents with grade Ⅳ obesity undergoing bariatric surgery is increasing, and significant shifts have occurred in the choice of surgical techniques: the use of Roux-en-Y gastric bypass (RYGB) and adjustable gastric banding (AGB) has declined markedly, while LSG has replaced the former as the predominant surgical approach.^10^^,^^11^ However, there is currently a lack of analysis on the short-term efficacy of LSG in adolescents with grade Ⅳ obesity. Grade Ⅳ obesity in adolescents is characterized by a high baseline body weight and extensive intra-abdominal fat accumulation, which compromises surgical exposure, limits instrument maneuverability within the abdominal cavity, increases operative difficulty compared to patients with general obesity, and prolongs operative time.^12^ Therefore, this study aims to investigate the effects of LSG on metabolic parameters and surgical safety in adolescents with grade Ⅳ obesity.

## Method

### *Study design and participants*

This study enrolled 31 patients from the Department of Bariatric and Metabolic Medicine, the First Hospital of Hebei Medical University, between May 2023 and September 2024, based on inclusion and exclusion criteria, and their clinical data were analyzed. All patients underwent bariatric surgery according to the guidelines for indications of metabolic and bariatric surgery issued by the International Federation for the Surgery and Other Therapies for Obesity (IFSO) and the American Society for Metabolic and Bariatric Surgery (ASMBS). Inclusion criteria: 1) Aged 14–19 years; 2) BMI ≥ 50 kg/m^2^; 3) Poor response to preoperative conservative weight-loss therapy; 4) No surgical contraindications identified during preoperative examinations; 5) Complete clinical data. Exclusion criteria: 1) Secondary obesity; 2) Severe psychiatric disorders; 3) Inability to comply with postoperative dietary and lifestyle modifications, indicating poor adherence; 4) History of major upper abdominal surgery. This observational study was conducted and reported in compliance with the STROBE Statement.

### *Preoperative preparation and surgical methods*

**Fig. 1:**
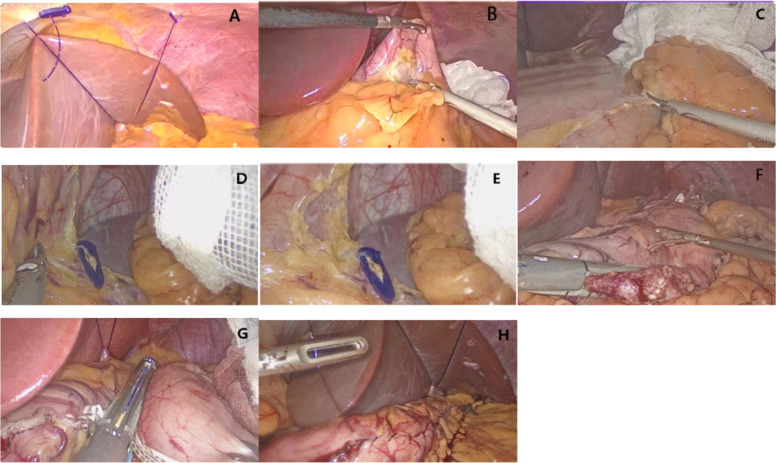
The main surgical steps of LSG. (A) Elevate the liver to expose the esophagogastric junction. (B) Mobilization begins 4 cm proximal to the pylorus. (C) Mobilize the greater omentum along the gastric wall. (D) Divide the short gastric vessels. (E) Dissect up to the angle of His to expose the left crus of the diaphragm. (F) The first stapler firing is performed 4 cm above the pylorus, dividing the gastric tissue along the greater curvature. (G) The final stapler firing is performed along a 36 Fr bougie, dividing the gastric fundus 1.5 cm away from the angle of His. (H) Reinforce the staple line of the gastric remnant with a continuous seromuscular suture.

Preoperatively, all adolescent patients underwent multidisciplinary evaluation with no contraindications detected. All LSG procedures were performed by experienced bariatric surgeons via a standard three-port technique (Fig. 1).

### *Postoperative management and follow-up*

After surgery, patients were managed by an experienced medical team, who closely monitored for procedure-related complications such as gastric leakage and bleeding. Early ambulation and adequate fluid intake were encouraged. A case manager provided patients with guidance on diet, physical activity, and follow-up.

### *Statistical methods*

Continuous variables are expressed as mean ± standard deviation ($\bar{x}\pm s$). Paired samples *t*-tests were used for comparisons of continuous variables (measurement data) before and after surgery. A p-value < 0.05 was considered statistically significant. GraphPad Prism 10.0 was utilized for graph generation, and SPSS 25.0 was employed for statistical analysis of the data.

## Result

### *General information*

In total, all 60 patients successfully completed laparoscopic surgery without conversion to open surgery or perioperative death. At the 1-year postoperative follow-up, 31 patients completed follow-up as scheduled, 21 patients were lost to follow-up, and 9 patients withdrew their informed consent. Preoperative baseline characteristics, complications, and preoperative use of GLP-1 receptor agonists in all patients (Table 1). No short-term complications such as infection, gastric leakage, bleeding, or deep vein thrombosis occurred postoperatively. The mean operative time was (90.23 ± 28.54) minutes, and the mean intraoperative blood loss was (50.43 ± 20.43) mL. At 12-months postoperatively, 31 patients completed regular follow-up. One case of fat liquefaction was observed, which resolved after conservative treatment, with no other long-term complications requiring intervention. Preoperatively, 12 patients had hypertension, 8 had type 2 diabetes mellitus, 4 had Polycystic Ovary Syndrome (PCOS), and 10 had NAFLDS. At 1-year after surgery, all patients with hypertension and type 2 diabetes mellitus achieved remission criteria. In addition, imaging findings and liver function improved significantly in 7 of the 10 patients with fatty liver disease, and clinical manifestations improved in 3 of the 4 patients with PCOS.

**Table 1:** Baseline demographic and clinical characteristics of patients stratified by previous use of GLP-1 analogues.

Characteristic	*n* = 31
Age (years)	16.13 ± 1.32
Female (%)	(17) 54.84
Height (m)	168.48 ± 7.58
Weight (kg)	154.13 ± 19.36
BMI (kg/m^2^)	51.94 ± 3.82
T2D (%)	8 (25.81)
Hypertension (%)	12 (38.71)
HbA1c (%)	5.11 ± 1.31
Previous use of GLP-1 analogue (%)	
Yes	3(9.68)
No	28(90.32)
Sermaglutide dose (mg)	0.5
Duration of Sermaglutide use (month)	3.11 ± 1.01

### *Postoperative obesity improvement in adolescent patients with grade IV obesity*

**Fig. 2:**
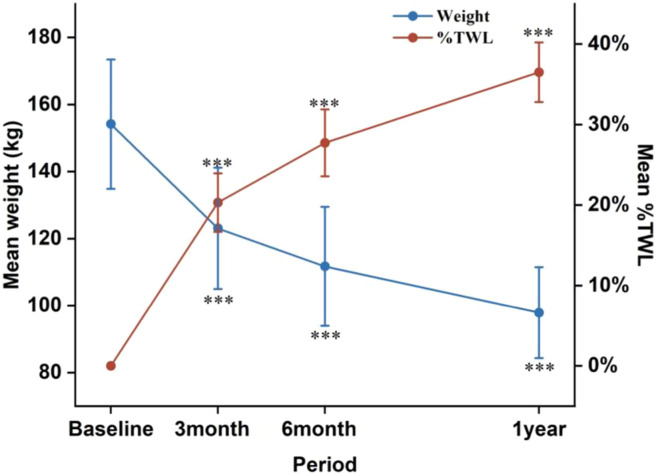
Postoperative Weight Reduction. Error bars represent standard deviation. * *P* < 0.05, ** *P* < 0.01, *** *P* < 0.001, all compared with the baseline value at each follow-up time point. %TWL indicates total weight loss percentage.

The body weight of all patients decreased from a baseline of (154.13 ± 19.31) kg to (123.04 ± 17.83) kg at 3-months postoperatively, (111.71 ± 17.72) kg at 6-months postoperatively, and (97.88 ± 13.65) kg at 1-year postoperatively. The corresponding %TWL was 23.01 ± 3.64% at 3-months, (27.73 ± 4.16) % at 6-months, and (36.49 ± 3.71) % at 1-year postoperatively (Fig. 2).

### *Glycemic metabolism indicators*

Fasting blood glucose showed no significant decrease during postoperative follow-up. Hemoglobin A1c (HbA1c) decreased significantly within 6-months postoperatively, though no significant difference was observed at the 3-month follow-up. Homeostatic Model Assessment for Insulin Resistance (HOMA-IR) demonstrated a significant decreasing trend throughout the 12-month postoperative follow-up period (Table 2).

**Table 2:** Changes in metabolic parameters before and after laparoscopic sleeve gastrectomy.

Indicator	Preoperation	3-month postoperatively	6-month postoperatively	1-year postoperatively
FBG (mmoL/L)	4.92 ± 1.62	4.62 ± 0.74	4.71±0.52	4.52±0.63
HbA1c (%)	5.11 ± 1.31	4.35 ± 0.82**	4.13±0.33**	4.63±0.45*
HOMA-IR	57.49 ± 23.64	17.33 ± 1.84**	15.17±5.92**	15.58±6.21**
ALT (U/L)	64.82 ± 33.2	26.71 ± 6.64**	25.13±7.81**	28.64±7.22**
AST (U/L)	40.26 ± 13.83	23.61 ± 9.02**	17.63±2.92**	16.92±2.61**
UA (µmoL/L)	491.5 ± 92.41	440.51±53.21**	360.83±20.22**	376.84±23.63**
RBC (×10^12^/L)	4.75±0.21	5.11±0.16	4.91±0.24	4.92±0.15
HB(g/L)	148.73±12.21	139.32±7.91*	135.41±8.97**	147.96±8.28
PLT (×10^12^/L)	289.76±18.96	272.22±15.53*	295.31±12.92*	302.15±10.01**
VitB_12_ (pg/mL)	268.07±87.50	238.12±29.82**	262.93±31.5	271.62±11.73
VitD (nmoL/L)	87.12±13.09	73.12±12.77*	81.42±12.63	89.07±12.99*
Ca (mmoL/L)	1.31±0.19	1.19±0.16*	1.33±0.11	1.35±0.11
Fe (mmoL/L)	8.92±1.14	7.64±0.99*	8.29±0.83**	9.23±0.67

Values in bold indicate statistically significant differences; * indicates *P* < 0.05, ** indicates *P* < 0.01.

### *Lipid metabolism indicators*

**Fig. 3:**
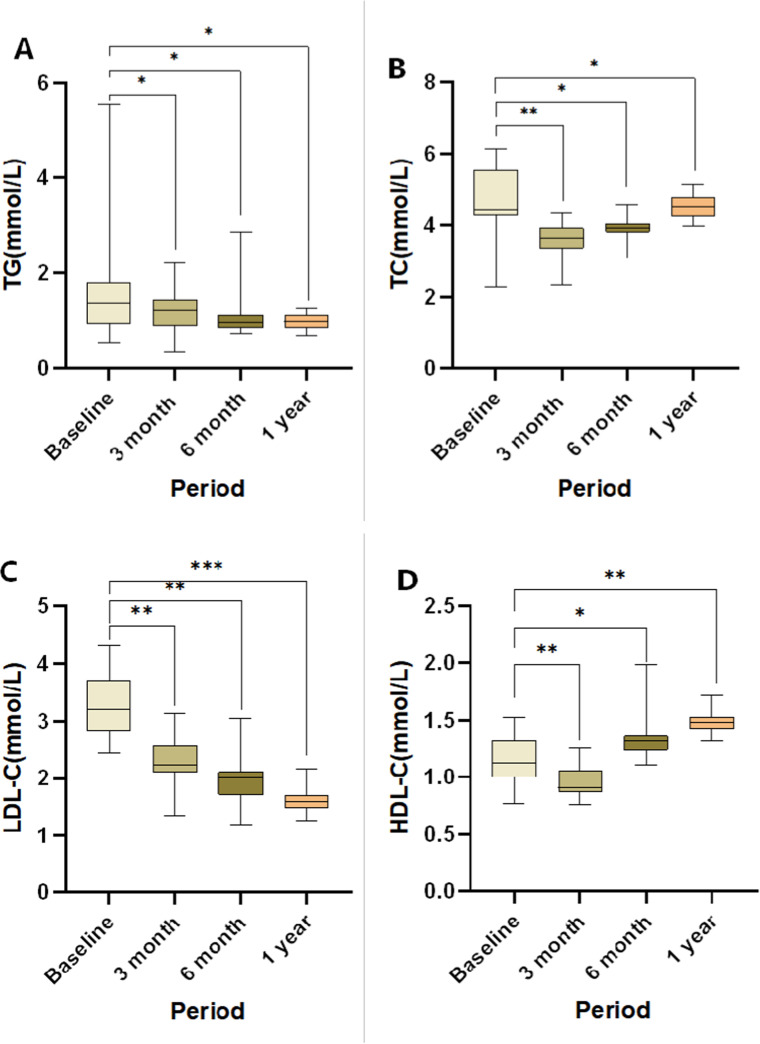
Postoperative Lipid Metabolism Profile (A) TG: Triglycerides; (B) TC: Total Cholesterol; (C) LDL-C: Low-Density Lipoprotein Cholesterol; (D) HDL-C: High-Density Lipoprotein Cholesterol. * *P* < 0.05, ** *P* < 0.01, *** *P* < 0.001 compared with preoperative values during postoperative follow-up.

Compared with preoperative levels, the levels of Total Cholesterol (TC), Triglycerides (TG), and Low-Density Lipoprotein Cholesterol (LDL-C) in patients were significantly reduced within one year postoperatively (*p* < 0.05). The level of High-Density Lipoprotein Cholesterol (HDL-C) showed a decrease within the first 3 postoperative months but gradually recovered and exhibited a sustained upward trend in subsequent follow-ups (*p* < 0.05) (Fig. 3).

### *Liver function*

Both ALT and AST levels were above the normal range preoperatively, indicating liver dysfunction associated with Grade IV obesity. These levels were normalized by the 3rd postoperative month and showed a significant decrease within one year postoperatively (*p* < 0.05). Uric acid levels also significantly decreased within one year (*p* < 0.05) (Table 2).

### *Hematological parameters*

Hemoglobin showed a decreasing trend within 6-months postoperatively (*p* < 0.05), while platelet counts demonstrated a decreasing trend within 3-months postoperatively (*p* < 0.05). Both parameters remained within the normal range (Table 2).

### *Postoperative nutritional status*

Iron (Fe) levels decreased within 6-months postoperatively. Levels of Vitamin B12 (VitB12), Vitamin D (VitD), and Calcium (Ca) decreased within 3-months postoperatively (*p* < 0.05). All values remained within the normal reference range (Table 2).

## Discussion

This study retrospectively analyzed the safety and efficacy of LSG in 31 adolescents with grade Ⅳ obesity over a 1-year follow-up. At 1 year postoperatively, the percent total weight loss (%TWL) reached 36.49%, and BMI decreased by 21.68 kg/m². Except for one case of fat liquefaction, no other complications were observed. These findings confirm the short-term safety and efficacy of LSG in this high-risk population of adolescents with grade Ⅳ obesity. For patients with severe obesity, bariatric surgery is a clinically effective and cost-efficient treatment modality.^13^ In terms of surgical mechanism, LSG works primarily by resecting approximately 80% of the stomach along the greater curvature. In contrast, RYGB involves creating a small gastric pouch and rerouting the intestinal tract, allowing nutrients to bypass the remnant stomach, duodenum, and proximal jejunum.^14^ Currently, LSG is the most commonly performed bariatric procedure worldwide due to its relative technical simplicity, favorable perioperative safety, reliable weight-loss efficacy, and significant benefits in improving metabolic comorbidities.^15^ After RYGB, changes in gut microbiota composition, reduced endotoxin levels, and alleviated systemic inflammation may contribute to sustained weight loss and improved metabolic syndrome.^16^, ^17^, ^18^ A prospective study reported that adolescents achieved a %TWL of 29% and a BMI reduction of 16.9 kg/m² five years after RYGB, while those who underwent LSG attained a %TWL of 27% and a BMI reduction of 15.0 kg/m² at a five-year follow-up.^19^ In a randomized controlled trial by Kajsa et al ^20^, 50 patients with severe obesity were randomly assigned to either a bariatric surgery (BS) group or an intensive non-surgical treatment. At 2-year follow-up, the BS group showed a BMI decrease of 12.6 kg/m² compared with only 0.2 kg/m² in the non-surgical group. One case of symptomatic gallstone formation occurred in the surgery group, and no significant nutritional differences were observed between groups.

Obesity markedly increases the prevalence of non-alcoholic fatty liver disease (NAFLD), which can progress to hepatocellular carcinoma (HCC)and substantially elevate mortality.^21^ Bariatric surgery promotes weight loss while reducing hepatic inflammation, fat accumulation, and fibrosis.^22^ NAFLD can be diagnosed using a combination of serologic markers (ALT, AST, lipid profile) and imaging (abdominal ultrasound or CT). However, liver biopsy is indicated for patients at high risk of steatohepatitis or fibrosis.^23^ In this study, patients did not meet the criteria for liver biopsy. Within the 1-year follow-up, ALT and AST decreased significantly (*P* < 0.05), and abdominal CT showed resolution or improvement of hepatic steatosis, with a remission rate of 75%. Obesity impairs the cholesterol efflux capacity (CEC) of HDL-C, increasing cardiovascular and cerebrovascular events. However, sleeve gastrectomy (SG) can improve CEC and reduce cardiovascular risk in obese patients.^24^^,^^25^ In this study, TG, TC, and LDL-C decreased significantly postoperatively (*P* < 0.05).

Adolescents with grade Ⅳ obesity have a much higher risk of T2DM than those with mild obesity. The prevalence of T2DM in severely obese adolescents ranges from 22% to 36%, and severe obesity is the strongest predictor of diabetes onset.^26^^,^^27^ Furthermore, adolescents show more rapid progressive β-cell dysfunction than adults with type 2 diabetes.^28^ Simultaneously, adolescent-onset T2DM is associated with earlier onset, more severe complications such as microalbuminuria and retinopathy, and reduced responsiveness to hypoglycemic medications.^29^^,^^30^ Although RYGB and LSG provide similar weight-loss efficacy, some studies suggest that RYGB has a more potent effect on T2DM, mainly because rapid nutrient entry into the intestine stimulates GLP-1 and PYY secretion, enhancing insulin release and sensitivity.^31^ Complete T2DM was defined as fasting glucose < 5.6 mmol/L and an HbA1c level < 6.5% off medication. Partial remission of type 2 diabetes was defined as a fasting glucose level of 5.6–6.9 mmol/L and an HbA1c < 6.5%.^25^ In this study, the postoperative improvement in blood glucose levels was not pronounced, which may be attributed to the limited number of type 2 diabetes patients and their generally low baseline blood glucose levels. However, a significant improvement was observed in HOMA-IR — a core indicator of insulin resistance — along with a 100% complete remission rate of type 2 diabetes. Because LSG alters the normal gastrointestinal anatomy and nutrient absorption capacity, weight loss may concurrently increase the risk of nutritional deficiencies.^32^ The proportion of patients with preoperative GLP-1 analogue exposure was low (9.68%), which had a limited impact on the short-term efficacy and safety outcomes of LSG in this cohort.

At 3-month follow-up, vitamin D, vitamin B12, calcium, and iron decreased significantly (*P* < 0.05) but remained within normal ranges. Notably, Fe levels at 6 months remained below baseline. These findings are consistent with meta-analyses reporting nutritional deficiency risks after adolescent bariatric surgery.^33^ Although no values fell below the lower limit of normal and a recovery trend was observed, strict nutritional monitoring, multivitamin and mineral supplementation, and targeted iron and calcium replacement remain essential for adolescents in a critical growth and development period. This study has several limitations, including a relatively small sample size and short follow-up duration, which limit the generalizability of the findings and prevent assessment of long-term efficacy, safety, and nutritional sustainability. The anxiety screening revealed high levels of anxiety in many participants. Future research should explore psychological outcomes, evaluate the impact of bariatric surgery on skeletal development, and compare different surgical techniques. Larger studies with longer follow-up are needed to provide stronger clinical evidence for this population.

## Conclusion

LSG is a safe and effective surgical intervention for severe obesity in adolescents, achieving significant weight loss in the short term and effectively improving metabolic syndrome and related complications. However, close attention and systematic management of postoperative nutritional status are essential to prevent micronutrient deficiencies. For adolescents with grade V obesity who meet the surgical criteria, LSG should be considered an important therapeutic option.

## Data availability statement

All data generated or analyzed during this study are included in this article.

## Authors’ contributions

GJF designed the study. WGQ and ZJ contributed to the data collection date analysis. PLY and GHB participated in data interpretation, literature research, and preparation of the manuscript. All authors read and approved the manuscript.

## Ethics approval and consent to participate

This study has been approved by the Ethics Committee of the First Hospital of Hebei Medical University (n°2025yanshen-196). All participants signed an informed consent.

## Consent for publication

Not applicable.

## Funding

Not applicable.

## Declaration of competing interest

The authors declare no conflicts of interest.
